# The knowledge and attitude about HIV/AIDS among Jordanian dental students: (Clinical versus pre clinical students) at the University of Jordan

**DOI:** 10.1186/1756-0500-4-191

**Published:** 2011-06-15

**Authors:** Soukaina T Ryalat, Faleh A Sawair, Mohammad H Shayyab, Wala M Amin

**Affiliations:** 1 Department of Oral and Maxillofacial Surgery, Oral Medicine, Oral pathology and Periodontology, Faculty of Dentistry, University of Jordan. Amman, Jordan; 2 Department of Prosthetic Dentistry, Faulty of Dentistry, University of Jordan

**Keywords:** Awareness, Dental students, Human immunodeficiency virus (HIV), Jordan

## Abstract

**Background:**

The present study aimed to address the suspected deficiency in the level of understanding of HIV/AIDS among clinical and pre clinical dental students at the University of Jordan. In this cross-sectional study, structured questionnaires were distributed to fifth year dental students (n = 121) and to third year dental students (n = 144) in the academic year 2008/2009.

**Findings:**

Significantly higher percentage of fifth-year students compared to third-year students felt that the teaching they received on cross-infection precautions and barrier dentistry was adequate (P < 0.001). Majority (84.2%) of fifth-year students were aware that individual carrying anti-HIV antibodies to be an HIV carrier, only 57.7% of third-year students were aware of this fact (P < 0.001). Majority recognized the association between Kaposi sarcoma, oral candidiasis and hairy leukoplakia with HIV/AIDS but knowledge of the association between HIV/AIDS with less frequent lesions was inadequate.

Significantly higher proportion of third-year students compared to fifth-year (39.2% vs. 26.3%) thought that HIV patients should be referred to other centers or support groups for treatment (P = 0.04).

**Conclusions:**

The level of knowledge of Jordanian dental students about HIV and AIDS was generally acceptable; there were inadequacies, however, in their understanding regarding some aspects of AIDS epidemic which demands that dental school curriculum needs some improvement.

## Introduction

The AIDS epidemic is continuing to grow [1]; global estimates indicated that over 40 million people are infected [2]. The fact that the number of HIV-infected patients under dental care is expected to increase [3] highlights the importance of providing healthcare, part of which dental treatment, to all individuals indiscriminately [4]. The reports indicated that about 90% of the HIV infections among healthcare workers occur in developing countries where occupational safety is a neglected issue [5-7].

Jordan has low HIV/AIDS prevalence. In 2007, there were an estimated 380,000 people living with HIV/AIDS in the region, according to UNAIDS. Although figures are low compared with southern Africa or Asia, they are still a cause for alarm, particularly since they are rising rapidly, especially among high-risk groups [8].

The human immunodeficiency virus (HIV) is the virus that causes AIDS. HIV attacks the immune system by destroying CD4 positive (CD4+) T cells. The acquired immunodeficiency syndrome (AIDS) is the end stage of HIV infection. A person infected with HIV is diagnosed with AIDS when he or she has one or more opportunistic infections, such as pneumonia or tuberculosis, and has a dangerously low number of CD4+ T cells less than 200 cells per cubic millimeter of blood [9]. All dental students should have complete knowledge about the universal precautions which is an administrative control measure that calls for the implementation of practices and equipment to protect the health care workers whenever the potential exists for exposure to blood. Every patient is considered to be infected with a blood-borne pathogen regardless of the known sero-status [10].

Clinical dental students in particular may encounter a number of incidents where infections, including AIDS, from patient's body fluids may occur. In comparison to other infectious diseases, dental students were found to be more willing to treat HBV- and HCV-infected patients than those with HIV infection [11]. Male students were reported to have significantly stronger negative attitudes towards patients at risk for or with HIV infections/AIDS than female students [12]. Dental students were reported to favor the inclusion in their tutored programs a more comprehensive material about patients with HIV infections/AIDS, such materials as case studies, discussion groups and closely supervised clinical experiences [12].

The dental school at the University of Jordan though not the largest in the Middle East but one of the accredited few. The school provides five-year training programs for about 800 students among them there are nearly 300 clinical students who carry out a comprehensive training in a well structured two-year clinical program. This program covers studying and training in the various disciplines of clinical dentistry and during which, it is estimated that about 30000 patients receive appropriate dental treatment every year. Such frequent exposure of the clinical students to patients entails a solid theoretical background on the part of the students of the infectious diseases and their means of transmission. In the first three years in dentistry, students mainly study basic science, in the third year they study virology which includes lectures about HIV, while clinical sessions and treating patients starts in the 4^th ^academic year. So the different clinical aspects related to infectious diseases are dealt with during 4^th ^and 5^th ^academic years, where by at the end of 5^th ^year, students should have completed the courses of oral pathology, oral medicine and oral surgery that relate to infectious diseases.

The dental literature is replete with published reports that evaluated the knowledge of clinical dental students of HIV/AIDS and their attitude towards infected patients with this disease [1, 11, and 13]. The authors are unaware, however, of any similar investigation that had been carried out on the dental students in Jordan.

Owing to the alarmingly increasing spread of HIV in the Middle East, the present study aimed to address the suspected deficiency in the level of understanding and awareness of this disease among clinical dental students at the University of Jordan. The study was planned to evaluate the clinical students' knowledge of HIV infection, virology, routs of transmission and cross infection methods and to compare it with the third year pre clinical dental students.

## Materials and methods

A structured questionnaire was distributed to all third year (144 students) and to all final year dental students (121 students); the sample size was fixed, at the end of obligatory lectures in the academic year 2008/2009 after the approval of the department at the faculty, to assess their knowledge of and attitude towards HIV/AIDS. The questionnaire covered the following aspects (see additional file 1); perception on the adequacy of curriculum preparation on HIV/AIDS which included 5 items using 4 response categories ranging from more than adequate to no teaching received; knowledge on HIV included 4 items using 2-5 responses where there is only one correct response, knowledge about associated lesions included 8 items using 4 response categories ranging from the right answer to don't know, knowledge of potential transmission routes of HIV included 8 items using 2 responses either yes or no, and students' attitude and practice behavior which included one item using 2 responses of either to refer or treat these patients. The questionnaire was similar to that used by Gilbert and Nuttall [13]. With the exception of age and sex no additional socio-demographic variables were collected and available for analysis.

Dentistry at Jordan University includes 5 academic years, third year is the final basic year, and 5th year is the final basic and clinical year. So comparison was made between them. Third year students study virology, having no clinical contact with patients, while 5^th ^year students treat patients, and should have covered the whole subject related to infectious diseases related to dental work, specifically HIV.

Statistical analysis was performed using SPSS for Windows release 17.0 (SPSS Inc., Chicago, IL, USA). Frequency distributions were obtained and chi-square test was used to compare differences between groups. Statistical significance was set at p < 0.05.

### Findings

Ninety seven (16 males, 81 females) third-year students out of 144 (67.4%) and 114 (27 males and 87 females) fifth-year students out of 121 (94.2%) completed the questionnaires. The age range of the third-year students was 19-22 (mean 20.6 ± 0.55 year) and that of fifth-year student was 22-24 years (mean 22.7 years ± 0.56 year).

### Adequacy of curriculum (Table 1)

**Table 1:** Third- and fifth-year Jordanian dental students' evaluation of the teaching they had received on five topics

Topic	More than adequate (%)*		Adequate (%)*		Less than adequate (%)*		No teaching received (%)*		P value
		
	**3**^**rd **^**year** (n = 97)	**5**^**th **^**year** (n = 114)	**3**^**rd **^**year** (n = 97)	**5**^**th **^**year** (n = 114)	**3**^**rd **^**year** (n = 97)	**5**^**th **^**year** (n = 114)	**3**^**rd **^**year** (n = 97)	**5**^**th **^**year** (n = 114)	
Cross-infection precautions	6.2	26.3	33.0	60.5	46.4	13.2	14.4	0	< 0.001
Virology	27.8	2.6	60.8	63.2	11.3	34.2	0	0	< 0.001
Sterilization practice and procedures	8.2	5.3	32.0	47.4	55.7	47.4	4.1	0	0.027
Barrier dentistry	8.2	26.3	44.3	65.8	32.0	7.9	15.5	0	< 0.001
Recognition of blood-borne virus risk group	18.6	10.5	57.7	55.3	22.7	34.2	1.0	0	0.11

(%)*: % of students who responded chose this option.

Significantly higher percentage of fifth-year students compared to third-year students felt that the teaching they received on cross-infection precautions and barrier dentistry as adequate (P < 0.001). In contrast, significantly higher percentage of fifth-year students (34.2%) compared to third-year students (11.3%) rated their teaching on virology as not sufficient to meet the need (P < 0.001) and, although the difference was statistically insignificant, higher percentage of fifth-year students (34.2%) compared to third-year students (23.7%) felt that the teaching they received on recognition of blood-borne virus risk groups as inadequate. The teaching received on sterilization practice and procedures was rated as inadequate by 59.8% of third-year students and 47.4% of fifth-year students (P = 0.027).

### Knowledge on HIV (Table 2)

**Table 2:** Third- and fifth-year Jordanian dental student's responses to three questions dealing with their academic knowledge

Questions	Options	(%) of students who responded with yes		
		
		3^rd ^year (n = 97)	5^th ^year (n = 114)	P value
Which host defense cells are primarily affected in AIDS?	Macrophages	7.2	0	< 0.001
	B-lymphocytes	11.3	2.6	
	Phagocytes	1.0	0	
	T-lymphocytes	75.3	97.4	
	Do not know	5.2	0	
If an individual is demonstrated to carry anti-HIV antibodies are they?	Definitely suffering from AIDS	9.3	2.6	< 0.001
	Immune to HIV infection	26.8	7.9	
	An HIV carrier	57.7	84.2	
	Do not know	6.2	5.3	
What is the average time interval between contracting HIV and the production of antibodies to it?	Less than 6 weeks	10.3	7.9	< 0.001
	6-12 weeks	58.8	21.1	
	13-24 weeks	11.3	7.9	
	24 weeks-5 years	10.3	15.8	
	Do not know	9.3	47.4	

The host defense cells primarily affected in AIDS were T lymphocytes according to 97.4% of fifth-year students and 75.3% of third-year students (P < 0.001). While the majority (84.2%) of fifth-year students were aware that an individual carrying anti-HIV antibodies to be an HIV carrier, only 57.7% of third-year students were aware of this fact (P < 0.001). Disappointingly, 7.9% of fifth-year students and 26.8% of third-year students thought that carrying anti-HIV antibodies means immunity against HIV infection. Surprisingly, only 11.3% of third-year and 7.9% of fifth-year students knew the time period between a person's first contact with the virus (infection) and the detection of HIV antibodies in blood or other fluids. There were significant proportions of respondents who thought it may take up to 5 years for these antibodies to be detectable.

### Knowledge of lesions associated with HIV/AIDS (Table 3)

**Table 3:** Third- and fifth-year Jordanian dental students' knowledge of lesions and conditions associated with HIV

Lesion or condition	Virtually exclusive to HIV (%)*		Associated with HIV in some cases (%)*		Unassociated with HIV (%)*		Do not know (%)*		P value
		
	3^rd ^year (n = 97)	5^th ^year (n = 114)	3^rd ^year (n = 97)	5^th ^year (n = 114)	3^rd ^year (n = 97)	5^th ^year (n = 114)	3^rd ^year (n = 97)	5^th ^year (n = 114)	
Oral Kaposi sarcoma	56.7	21.1	37.1	73.7	2.1	0	4.1	5.3	< 0.001
Oral candidiasis	4.1	5.3	87.6	94.7	5.2	0	3.1	0	0.02
Oral hairy leukoplakia	24.7	15.8	66.0	84.2	2.1	0	7.2	0	0.002
Salivary gland enlargement	2.1	0	39.2	65.8	38.1	15.8	20.6	18.4	< 0.001
Xerostomia	2.1	0	22.7	76.3	38.1	23.7	37.1	0	< 0.001
Oral melanotic hyperpigmentation	0.0	2.6	10.3	36.8	29.9	34.2	59.8	26.3	< 0.001
ITP	3.1	0	12.4	50.0	28.9	23.7	55.7	26.3	< 0.001
Crohn's disease	2.1	2.6	13.4	13.2	30.9	76.3	53.6	7.9	< 0.001

(%)*: % of students who responded with a yes to each option. ITP: idiopathic thrombocytopenic purpura

The association of Kaposi sarcoma, oral candidiasis, and oral hairy leukoplakia with HIV was known by higher proportion of fifth-year compared to third- year students. However, some students believed that these lesions were virtually exclusive to HIV/AIDS; 56.7% of third-year students thought that Kaposi sarcoma is exclusive to HIV/AIDS. Approximately one-third of fifth-year students and majority of third-year students were unaware of the association between salivary gland disease and HIV/AIDS. A significant proportion of the students, particularly third-year, did not link or know the association between oral melanotic hyperpigmentation and idiopathic thrombocytopaenic purpura with HIV/AIDS. Of the third-year students surveyed, less than one-third knew that Crohn's disease and HIV are unassociated compared to three-quarters of fifth-year students who knew this information.

### Knowledge of potential transmission routes of HIV (Table 4)

**Table 4:** Third- and fifth-year Jordanian dental students' view on potential transmission routes of HIV

Potential transmission routes of HIV	(%) of students who responded yes		
	
	3^rd ^year (n = 97)	5^th ^year (n = 114)	P value
Your unbroken skin in contact with unbroken skin of HIV-positive patient	18.6	15.8	0.59
Your unbroken skin in contact with blood of HIV-positive patient	32.0	34.2	0.73
Your unbroken skin in contact with saliva of HIV-positive patient	22.7	34.2	0.07
Your cut skin in contact with unbroken skin of HIV-positive patient	25.8	28.9	0.61
Your cut skin in contact with blood of HIV-positive patient	93.8	100	0.007
Your cut skin in contact with saliva of HIV-positive patient	84.5	81.6	0.57
Inhalation of aerosol containing blood of HIV-positive patient	73.2	73.7	0.94
Inhalation of aerosol containing saliva of HIV-positive patient	61.9	44.7	0.01

Some students, without significant difference between clinical- and pre clinical-year students, thought that HIV can be transmitted to them if their unbroken skin comes into contact with unbroken skin, saliva, or blood of an HIV-positive patient. Although all fifth-year and 93.8% of third-year students recognized that contact of a cut skin with blood of HIV-positive patient represented a transmission risk, majority of clinical and pre clinical students considered contact of a cut skin with saliva of HIV-positive patient and nearly one-quarter of them thought that contact of a cut skin with unbroken skin of HIV-positive patient as potential transmission routes of HIV. Regardless of educational level, inhalation of aerosol was considered as means of cross-infection by nearly three-quarters of students when the aerosol contains blood of HIV-positive patient, but significantly higher proportion of third-year students thought that aerosol containing saliva of HIV-positive patient could transmit HIV infection.

### Attitude and behavior practices towards HIV/AIDS patients

There was 73.7% of fifth-year students and 60.8% of third-year students who thought that HIV/AIDS patients should be treated at any dental facility with same respect and dignity as other patients but after taking special precautionary measures. Significantly higher proportion of third-year students compared to fifth-year (39.2% vs. 26.3%) thought that these patients should be referred to other centers or support groups for treatment (P = 0.04).

## Discussion

In this study, comparison was made between 3d and 5^th ^year dental students regarding their knowledge and attitude about HIV. The sample was homogenous; there were no ethnic or religious variations, future studies are encouraged to study the influence of other factors, which were not studied here such as the social class, the income, education of parents and the area of residency on the student's attitude towards HIV patients, as this is a limitation of this study. Also future studies are encouraged to hold this study among dental students of the other dental faculty in Jordan and among other health care workers.

Dentists have a responsibility to provide HIV-infected patients, particularly because oral lesions are common among these patients. It is obvious that having adequate knowledge about HIV/AIDS enhances confidence in student's ability to manage infected patients. In the present investigation most of the clinical dental students rated the teaching they received on cross-infection precautions and barrier dentistry as adequate or more than adequate. However, there was a lower degree of satisfaction expressed for instruction on sterilization practice and procedures and recognition of blood-borne virus risk groups especially among pre clinical students. In contrast, students showed a higher degree of satisfaction in the latter two teaching topics in a similar study conducted in UK [13]. A rather surprising finding of the present investigation indicated that only 11.3% of third-year and 7.9% of fifth-year students knew the average time period of 6-12 weeks [14] required for HIV seroconversion. This may explain the low satisfaction among students by the teaching they received in virology.

A significant number of students did not know when it is possible to confirm the HIV infection. Some students thought it takes up to five years for seroconversion to happen. Disappointingly, some students even thought that carrying anti-HIV antibodies indicates immunity against the disease. The inadequate knowledge of HIV virology was also reported by Nigerian [15] and Sudanese dental students [16].

Providing proper dental care to HIV/AIDS patients necessitate good knowledge in the recognition of the oral lesions associated with the disease. As many as 40 oral manifestations of HIV infection have been reported [17]. The results in this study showed adequate knowledge of the lesions strongly associated with HIV/AIDS such as Kaposi sarcoma, hairy leukoplakia, and oral candidiasis. However, the clinical and especially the pre clinical students needed a broader knowledge of lesions less strongly associated with HIV such as oral melanotic hyperpigmentation, idiopathic thrombocytopenic purpura, and salivary gland disease. Students should also be educated that even the lesions strongly associated with HIV/AIDS are not exclusive to HIV/AIDS. Kaposi sarcoma, hairy leukoplakia and oral candidiasis may also be seen in patients not having HIV infection or AIDS. However, the overall knowledge of the Jordanian dental students about the oral manifestations of HIV/AIDS is considered satisfactory compared to that of dental students in countries where AIDS is endemic [15].

Since oral lesions are common in HIV/AIDS patients, oral health care is an important component of their treatment plan. Although many dentists used to reject providing dental treatment to AIDS patients, dentists' attitudes toward the treatment of these patients have improved in recent years [18]. In the present study, only 39.2%(pre clinical) vs. 26.3% (clinical) of students were unwilling to treat HIV/AIDS patients and thought that they should be referred to other centers or support groups for treatment. The rest of the students thought that these patients should be treated at any dental center but felt that special precautionary measures are necessary during treatment. Although these results are in agreement with some published reports [12,19], the willingness of treating HIV/AIDS patients is, however, felt an over optimistic by enthusiastic students in this conservative society where AIDS is still in low prevalence rates. A study conducted in Jordan few years ago to investigate willingness of working dentists to treat HIV infected patients has shown that, when a fake AIDS patient contacted dental practices by phone for treatment of pain of dental origin, there was fear of the HIV illness and only 15% accepted to provide such treatment [20].

The fear of treating HIV-infected patients was further revealed by the inadequate knowledge of HIV transmission reported by the students participated in this study. Evidence indicated a low occupational risk for HIV infection among health care professionals [13]. The highest risk was following needle-stick injury and has been estimated as less than 0.5% per accident [21]. Exposure of broken skin to infected blood is a possible transmission route and has been considered by the vast majority of students. However, no evidence of HIV cross infection may follow exposure of unbroken skin to body fluids of HIV-infected patients [13].

Nearly one-quarter of the students thought that contact of a cut skin with unbroken skin of HIV-positive patient was a possible transmission route of HIV, further revealing the inadequate knowledge about HIV transmission, majority of them considered contact of a cut skin with saliva of HIV-positive patient could be a risk factor. But till this time there is no report of transmission of HIV from saliva alone Although HIV was isolated from saliva [13]. Inhalations of infected body fluids, such blood or saliva aerosols, could be considered as potential transmission routes of HIV [22], in this study, nearly three-quarter of the students thought that HIV infection could be transmitted through inhalation of aerosols containing HIV-infected blood, significantly higher proportion of the pre clinical students thought that aerosol containing saliva of HIV-positive patient could transmit HIV infection.

## Conclusion

The level of knowledge of Jordanian dental students (clinical and pre clinical) about HIV and AIDS was generally acceptable. There were, however, some inadequacies in the students' knowledge in some essential aspects of control and prevention of transmission of the disease. The dental school curriculum must, therefore, be updated and improved in order to enhance the students' knowledge in those aspects.

## Competing interests

The authors declare that they have no competing interests.

## Authors' contributions

SR carried out the study design participated in the sequence alignment and drafted the manuscript. MH carried out the data collection. FS performed the statistical analysis. WA supervised the work and helped to draft the manuscript. All authors read and approved the final manuscript.

## Supplementary Material

Additional file 1 **Questionnaire regarding final and third year dental student's knowledge of human immunodeficiency virus**. Final and third year Dental students' knowledge of human immunodeficiency Virus. Click here for file

## References

[B1] Cohen LA Romberg E Grace EG Barnes DM Attitudes of advanced dental education students toward individuals with AIDS J Dent Educ 2005 69 896 900 16081572

[B2] Ogunbodede EO Rudolf MJ Policies and protocol for preventing transmission of HIV infection in oral health care in South Africa S Afr Dent J 2002 57 469 474 12674867

[B3] Patton LL HIV disease Dent Clin North Am 2003 47 467 492 10.1016/S0011-8532(03)00016-8 12848460

[B4] Lohrmann C Valimaki M Suominen T Muinonen U Dassen T Peate I German nursing students' knowledge of and attitudes to HIV and AIDS: two decades after the first AIDS cases J Adv Nurs 2000 31 696 703 10.1046/j.1365-2648.2000.01326.x 10718890

[B5] Kermode M Holmes W Langkham B Thomas MS Gifford S Occupational exposure to blood and risk of bloodborne infection among health care workers in rural north Indian healthy care settings Am J Infect Control 2005 33 34 41 10.1016/j.ajic.2004.07.015 15685133

[B6] Ansa VO UdAnsa VO Udoma EJ Umoh MS Anah MU Occupational risk of infection by human immunodeficiency and hepatitis B viruses among health workers in south-eastern Nigeria East Afr Med J 2002 79 254 6 12638809 10.4314/eamj.v79i5.8863

[B7] Gumodoka B Favot I Berege ZA Dolmans WM Occupational exposure to the risk of HIV infection among health care workers in Mwanza Region, United Republic of Tanzania Bull World Health Organ 1997 75 133 40 9185365 PMC2486935

[B8] Epidemiological fact sheet on HIV and AIDS. Geneva: WHO/UNAIDS/UNICEF http://apps.who.int/globalatlas/predefinedReports/EFS2008/full/EFS2008_JO.pdf

[B9] HIV Infection and AIDS, An Overview, NIAID Fact Sheet: NIAID http://www.wrongdiagnosis.com/artic/hiv_infection_and_aids_an_overview_niaid_fact_sheet_niaid.htm

[B10] Centers for Disease Control Prevention Guidelines for infection control in health care personnel. Infect Control Hosp Epidemiol 1998 19 445

[B11] Hu SW Lai HR Liao PH Comparing dental students' knowledge of and attitudes toward hepatitis B virus-, hepatitis C virus-, and HIV-infected patients in Taiwan AIDS Patient Care STDS 2004 18 587 593 10.1089/apc.2004.18.587 15630786

[B12] Seacat JP Inglehart MR Education about treating patients with HIV infections/AIDS: the student perspective J Dent Educ 2003 67 630 640 12856963

[B13] Gilbert AD Nuttall NM Knowledge of the human immunodeficiency virus among final year dental students J Dent 1994 22 229 235 10.1016/0300-5712(94)90116-3 7962898

[B14] Centres for disease control Recommendations for preventing transmission of human immunodeficiency virus and Hepatitis B virus to patients during exposure prone invasive procedures MMWR Recomm Rep 1991 40 1 9 1648165

[B15] Ajayi YO Ajayi EO Dental students' knowledge of human immunodeficiency virus J Dent 2008 36 374 378 10.1016/j.jdent.2008.02.008 18336987

[B16] Nasir EF Astrøm AN David J Ali RW HIV and AIDS related knowledge, sources of information, and reported need for further education among dental students in Sudan--a cross sectional study BMC Public Health 2008 8 286 10.1186/1471-2458-8-286 18702806 PMC2527565

[B17] Samaranayake LP Oral care of the HIV patient Dent Update 1992 19 56 58 1291360

[B18] Senna MI Guimarães MD Pordeus IA Factors associated with dentists' willingness to treat HIV/AIDS patients in the National Health System in Belo Horizonte, Minas Gerais, Brazil Cad Saude Publica 2005 21 217 25 10.1590/S0102-311X2005000100024 15692655

[B19] Erasmus S Luiters S Brijlal P Oral Hygiene and dental student's knowledge, attitude and behaviour in managing HIV/AIDS patients Int J Dent Hyg 2005 3 213 217 10.1111/j.1601-5037.2005.00137.x 16451310

[B20] El-Maaytah M Al Kayed A Al Qudah M Al Ahmad H Moutasim K Jerjes W Al Khawalde M Abu Hammad O Dar Odeh N El-Maaytah K Al Shmailan Y Porter S Scully C Willingness of dentists in Jordan to treat HIV-infected patients Oral Dis 2005 11 318 322 10.1111/j.1601-0825.2005.01126.x 16120120

[B21] Cusini M Transmission of HIV infection Semin Dermatol 1995 14 202 204 10.1016/S1085-5629(05)80019-7 7488535

[B22] Blignaut E The role of the dental profession in the AIDS epidemic. Practitioners corner J Dent Assoc S Afr 1994 49 113 152 9508946

