# Supplementary material for: The knowledge and attitude about HIV/AIDS among Jordanian dental students: (Clinical versus pre clinical students) at the University of Jordan
Source: BMC Res Notes. 2011 Jun 15;4:191. doi: 10.1186/1756-0500-4-191 (PMC3127964; doi:10.1186/1756-0500-4-191)
Supplement: Additional file 1 — Questionnaire regarding final and third year dental student's knowledge of human immunodeficiency virus. Final and third year Dental students' knowledge of human immunodeficiency Virus. [file 1756-0500-4-191-S1.DOC]

**Final and third year Dental students’ knowledge of human immunodeficiency Virus**

□ **Student name: ----------------------** □ **Age:-----------yrs** □ **Gender: M F**

**A. Evaluation of teaching**

**What is your evaluation of the teaching you had received on the following topics?**

***Cross-infection precautions***

□ More than adequate □ Adequate □Less than adequate □ No teaching received

***Virology***

□ More than adequate □ Adequate □Less than adequate □ No teaching received

***Sterilization practice and procedures***

□ More than adequate □ Adequate □Less than adequate □ No teaching received

***Barrier dentistry* (e.g. Gloves, masks, protective eyewear)**

□ More than adequate □ Adequate □Less than adequate □ No teaching received

***Recognition of blood-borne virus risk group***

□ More than adequate □ Adequate □Less than adequate □ No teaching received

**C. Academic knowledge of HIV and AIDS**

- ***Tick only one answer of the following four questions you think it is correct:***

***1. Which host defence cells are primarily affected in AIDS?***

□ Macrophages □ B-lymphocytes □ Phagocytes

□ T-lymphocytes □ Do not know

***2. If an individual is demonstrated to carry anti-HIV antibodies are they?***

□ Definitely suffering from AIDS □ Immune to HIV infection

□ An HIV carrier □ Do not know

***3. What is the average time interval between contracting HIV and the production of antibodies to it?***

□ Less than 6 weeks □ 6–12 weeks □ 13–24 weeks

□ 24 weeks–5 years □ Do not know

4. Do you feel that HIV/AIDS is a problem in Jordan?

□ Yes □ No

**B. Knowledge of lesions and conditions associated with HIV**

**How could you describe the following lesions and conditions in association with HIV?**

***Oral Kaposi sarcoma***

□ Virtually exclusive to HIV □ Associated with HIV in some cases

□ Unassociated with HIV □ Do not know

***Oral candidiasis***

□ Virtually exclusive to HIV □ Associated with HIV in some cases

□ Unassociated with HIV □ Do not know

***Oral hairy leukoplakia***

□ Virtually exclusive to HIV □ Associated with HIV in some cases

□ Unassociated with HIV □ Do not know

***Salivary gland enlargement***

□ Virtually exclusive to HIV □ Associated with HIV in some cases

□ Unassociated with HIV □ Do not know

***Xerostomia***

□ Virtually exclusive to HIV □ Associated with HIV in some cases

□ Unassociated with HIV □ Do not know

***Oral melanotic hyperpigmentation***

□ Virtually exclusive to HIV □ Associated with HIV in some cases

□ Unassociated with HIV □ Do not know

***Idiopathic thrombocytopaenic pupurta***

□ Virtually exclusive to HIV □ Associated with HIV in some cases

□ Unassociated with HIV □ Do not know

***Crohns disease***

□ Virtually exclusive to HIV □ Associated with HIV in some cases

□ Unassociated with HIV □ Do not know

**D. Attitude and behaviour practices of students**

**1. *How and where do you think that HIV/AIDS patients should be treated?***

□ Should be referred to a support group

□ At any dental facility with the same respect and dignity as other patients after taking special precautionary measures

**E. View on potential transmission route of HIV**

**Do you think that there is cross-infection transmission risk when:**

***Your unbroken skin in contact with unbroken skin of HIV positive patient***

□ Yes □ No

***Your unbroken skin in contact with blood of HIV positive patient***

□ Yes □ No

***Your unbroken skin in contact with saliva of HIV positive patient***

□ Yes □ No

***Your cut skin in contact with unbroken skin of HIV positive patient***

□ Yes □ No

***Your cut skin in contact with blood of HIV positive patient***

□ Yes □ No

***Your cut skin in contact with saliva of HIV positive patient***

□ Yes □ No

***Inhalation of aerosol containing blood of HIV positive patient***

□ Yes □ No

***Inhalation of aerosol containing saliva of HIV positive patient***

□ Yes □ No
